# Hepatitis B surface gene variants isolated from blood donors with overt and occult HBV infection in north eastern Egypt

**DOI:** 10.1186/s12985-015-0389-y

**Published:** 2015-09-30

**Authors:** Rania Kishk, Nader Nemr, Abeer Elkady, Mohamed Mandour, Mohamed Aboelmagd, Nevene Ramsis, Mohamed Hassan, Nashaat Soliman, Sayuki Iijima, Shuko Murakami, Yasuhito Tanaka, Mostafa Ragheb

**Affiliations:** Department of Microbiology and Immunology, Faculty of Medicine, Suez Canal University, El Salam District, Ismaïlia, Egypt; Department of Endemic and Infectious diseases, Faculty of Medicine, Suez Canal University, El Salam District, Ismaïlia, Egypt; Department of Clinical and Chemical Pathology, Faculty of Medicine, South Valley University, Qena, Egypt; Department of Virology and Liver Unit, Nagoya City University Graduate School of Medical Science, Nagoya, 467-8601 Japan; Department of Clinical Pathology, Faculty of Medicine, Suez Canal University, El Salam District, Ismaïlia, Egypt; Department of Internal Medicine, Faculty of Medicine, Aljouf University, Sakaka, KSA Saudi Arabia

**Keywords:** Occult HBV, Genotype D, Escape mutation

## Abstract

**Background:**

Major hydrophilic region in genomic HBV extending from aa99 to aa169, clustered with a highly conformational epitope, is critical to the antigenicity of hepatitis B surface antigen (HBsAg) and may affect the diagnosis of HBV in HBV screening test. So, this study aimed to characterize variants of S gene product of hepatitis B virus (HBV) isolated from patients with overt or occult HBV infection in north-eastern Egypt.

**Methods:**

The study included sera of two different groups of volunteer blood donors (VBDs), 82 with overt HBV that were positive for HBsAg and anti-HBc and 343 donors negative for HBsAg eligible for donation. Of the latter group, only 44 were positive for anti-HBc. All anti-HBc positive sera were subjected to HBV DNA detection and partial sequence analysis targeting the HBV S gene.

**Results:**

HBV DNA was detected in 22.7 % of HBsAg-/anti-HBc + (10/44 patients) and in 90 % of HBsAg + donors (74/82 patients) with significant statistical difference (*P* = 0.0001). Phylogenetic analysis showed that HBV strains retrieved from both groups were of genotype D. Amino acid escape mutation T125M was detected in only 2 samples of the occult infection group and in none of the overt group (*P* = 0.01). Different amino acid substitutions were identified in overt infection group: S143L/T (16.2 %, 12/74) and P120T/S (2.7 %, 2/74). Q129R was significantly more frequent in cases with occult HBV infection (40 %, 4/10) than overt group (6.8 %, 5/74) (*P* = 0.01).

**Conclusions:**

HBV genotype D predominated both in patients with overt and occult HBV infection. Different profiles of amino acid substitutions in the major hydrophilic region were seen in these two groups in Egypt.

## Background

Hepatitis B virus (HBV) chronically infected 240 million people and leads to hepatitis, liver cirrhosis, and hepatocellular carcinoma. It is considered a major health problem in the world, especially in Asia, the Middle East, and Africa [1].

Hepatitis B surface antigen (HBsAg) is an envelope glycoprotein that serves as a primary target for diagnosis and immunoprophylaxis of HBV infection. The dominant epitopes of HBsAg, which are the targets of neutralizing antibody responses, reside in the “a” determinant (aa 124–147) within the major hydrophilic region (MHR). Amino acid substitutions in the MHR can cause reduced binding of anti-HBs antibodies, resulting in immune escape [2]. The major hydrophilic region, extending from aa99 to aa169, clustered with a highly conformational epitope, is critical to the antigenicity of HBsAg. Thus, amino acid substitution in the MHR, either from variants in natural isolates or mutants selected under immunological pressure, could cause incorrect diagnoses of HBV in the HBsAg screening test [3].

In Egypt, blood donation depends mainly on unpaid voluntary donors that could be either replacement or regular donors. Although, replacement donors could be either relatives or neighbors of blood recipients, the donated blood is used to replenish the stores of the blood bank rather to be given to a particular recipient except when rare blood groups are needed on emergency settings. Furthermore, selection of blood donors depends upon a screening system that excludes the subjects positive for HBsAg, anti-HCV, anti-HIV and anti-treponema antibody. Despite the importance of anti-HBc screening for safer blood transfusion [2, 3], this serological marker is not included in Egyptian blood bank screening. Then, such screening system in Egypt would miss occult HBV infection (OBI) among blood donors [2, 3].

Occult HBV infection (OBI) is defined as the long-term persistence of HBV DNA, despite undetectable HBsAg by regular immunoassays [4, 5]. Evidence from different geographical regions documents that OBI is globally distributed and potentially a major source of HBV transmission through blood transfusion and organ transplantation [6, 7]. In the Middle East, an estimated 2–5 % of the general population is chronically infected. Over the last two decades, , the endemicity of hepatitis B infection has been changed from high to intermediate and low endemicity the prevalence of hepatitis B is lower (2-6 %) [8–10].

HBV vaccination was introduced in Egypt in 1992 as part of an expanded program of immunization for the newborn, in addition to its availability for populations at high risk (Mansour et al., 1993) [11]. The incidence of OBI in Egypt was assessed by Attia [1998] [12]. In north eastern Egypt where the current study was conducted, Youssef and colleagues reported OBI in 35 (16.3 %) of 214 hospitalized patients with elevated ALT levels, while only 10 (4.7 %) were positive for HBsAg [13].

This study aimed to characterize HBs antigen MHR variants in cases with overt and occult HBV infection in volunteer blood donors (VBDs).

## Results

### General characteristics of the studied cohorts

The study included 126 volunteer blood donors, consisting of 44 HBsAg-negative/anti-HBc-positive men and 82 HBsAg-positive subjects (74 men and 8 women) (median viral load was 157.5660 × 10^3^ IU/m). Both groups were matched regarding age and ALT level (Table 1). Female gender was significantly more frequent in donors with overt HBV infection compared to HBV-resolved group (*P* = 0.049). A significantly higher frequency of detectable HBV DNA by nested PCR was observed in cases with overt HBV infection (74/82, 90 %) than in cases with occult HBV infection (10/44, 22.7 %) (*P* = 0.0001). Sequencing and phylogenetic analysis revealed that HBV sequences were of genotype D (data not shown).

**Table 1:** General characteristics of the studied cohort

	Total (*n* = 126)	HBsAg-/ anti-HBc+ (*n* = 44)	HBsAg+ (*n* = 82)	*P*-value
Age (Years) Mean ± SD	29.1 ± 7.1	32.9 ± 6.1	28.0 ± 7	NS
Gender (M)	118 (93.7)	44 (100 %)	74 (90 %)	0.049
ALT (IU/L)	18.8 ± 10.6	18.6 ± 13.9	19 ± 7.2	NS
HBV DNA S region (+)	84 (66.7 %)	10 (22.7 %)	74 (90 %)	0.0001
MHR Substitutions (%)	34/84 (40.5)	7/10 (50)^a^	27/74 (37.8)	0.5
T115S	7/84 (8.3)	0	7/74 (9.5)	NS
P120T/S	2/84 (2.3)	0	2/74 (2.7)	NS
T125M	2/84 (2.3)	2/10 (20)	0	0.01
P127T	1/84 (1.2)	1/10 (10)	0	NS
Q129R	9/84 (10.7)	4/10 (40)	5 (5.8)	0.01
K141R	1/84 (1.2)	0	1 (1.4)	NS
S143L/T	12/84 (14.3)	0	12 (16.2)	NS

^a^Two MHR mutants were present simultaneously in 2 cases; OCU18 and OCU158

### Genetic variants of HBsAg in the MHR

**Fig. 1:**
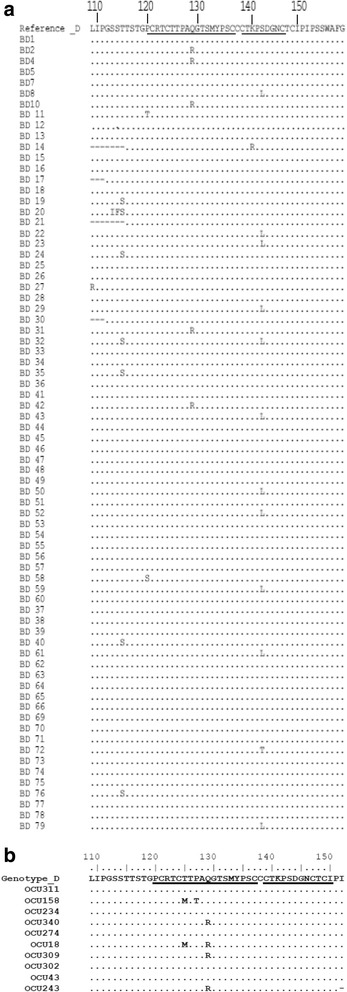
Alignment of amino acid sequences of the HBV partial surface gene encompassing the “a” determinant region isolated from cases with overt HBV infection **a** and from cases with occult HBV infection **b**. The detected sequences were aligned with respect to a consensus sequence of HBV genotype D retrieved from DDBJ/GenBank database. Dots in alignment indicate identity with the consensus sequence of genotype D. First and second loop positions are underlined in the consensus sequence of the genotype

In this study, MHR variants were found in 27/74 (37.8 %) of cases with overt HBV infection and 3 in 5/10 (50 %) of cases with occult HBV. Five types of variants were observed in donors with overt HBV infection. The most prevalent variant was S143L/T detected in 12 (16.2 %) of sequences, followed by T115S in 7 (9.5 %), Q129R in 5 (5.8 %), P120T/S in 2 (2.7 %), and K141R in one (1.4 %) (Fig. 1a) (Table 1). In 10 cases with occult HBV infection, a different profile of amino acid substitutions was observed. The most frequent one was Q129R in 4 (40 %) of the retrieved sequences, followed by T125M in 2 (20 %) and P127T in one (10 %) sample (Fig. 1b) (Table 1). The well-known G145A/R variant was not observed in either the overt infection or occult infection group. T125M and Q129R amino acid substitutions were significantly more frequent in cases with occult HBV infection than in overt HBV infection (2 and 4 of 10 vs. 0 and 5 of 74, *P* = 0.01 for each) (Table 1). The most prevalent variant was S143L/T detected in 16.2 % of sequences, followed by T115S in 9.5 %, Q129R in 5.8 %, P120T/S in 2.7 %, and K141R in 1.4 % (Fig. 1a) (Table 1).

Among overt HBV group (82 patients), 47 patients (57.3 %) had mild viremia (36 wild type/11 mutant type), 17 patients (20.7 %) had moderate viremia (9 wild type/8 mutant type), 3 patients (3.7 %) had high viremia (2 wild type/1 mutant type), and 12 patients (14.6 %) had very high viremia (7 wild type/5 mutant type). The remaining 8 samples that were negative by real time PCR and positive HBsAg were assumed to contain HBV DNA below the detection limit of the assay.

In cases with occult HBV infection, a different profile of amino acid substitutions was observed. The most frequent one was Q129R in 40 % (4/10) of the retrieved sequences, followed by T125M in 20 % and P127T in one sample (Fig. 1b) (Table 1). The well-known G145A/R variant was not observed in either the overt infection or occult infection group. T125M and Q129R amino acid substitutions were significantly more frequent in cases with occult HBV infection than in overt HBV infection (*P* = 0.01 for each) (Table 1).

### Criteria of blood donors with “HBsAg-/anti-HBc+”serological profile

According to positivity for anti-HBs, blood donors with serological profile [(HBsAg-/anti-HBc+)] were sub-classified into two groups; 17 cases positive for both anti-HBc and anti-HBs [(anti-HBc+/anti-HBs+)] and 27 positive for only anti-HBc [anti-HBc+/anti-HBs–)]. HBV DNA was detected in 10 samples, 5 in each group with a prevalence of 29.4 % in the [(anti-HBc+/anti-HBs+)] group, and 18.5 % in the [(anti-HBc+/anti-HBs-)] group. T125M was detected in one case in the anti-HBc+/anti-HBs + group (sample ID, OCU 18) and in one case in the anti-HBc+/anti-HBs– group (sample ID, OCU 158) (Fig. 1b) (Table 2). However, Q129R was observed more frequently in anti-HBc+/anti-HBs + group (3/5, 60 %) than in anti-HBc+/anti-HBs– group (1/5, 20 %), but this difference did not reach statistical significance.

**Table 2:** Characteristics of blood donors negative for HBsAg and positive for anti-HBc/anti-HBs

	Total (*n* = 44)	Anti-HBc positive/anti-HBs positive (*n* = 17)	Anti-HBc positive/anti-HBs negative (*n* = 27)	*P*-value
Age (Mean ± SD )	30 ± 7.1	32.3 ± 6.7	28.3 ± 7.2	0.055
ALT (U/L)	20.5 ± 15.9	20.9 ± 15.9	20.2 ± 16.2	NS
HBV DNA S region (+)	10 (22.7 %)	5 (29.4 %)	5(18.5 %)	NS
MHR substitutions				
T125M	2/10 (20 %)	1/5	1/5	NS
Q129R	4/10 (40 %)	3/5	1/5	NS

### Descriptive analysis of cases with occult HBV infection

HBV DNA was detected in 10 of 44 HBsAg-negative/anti-HBc-positive blood donors (22.7 %). The mean age of cases with occult HBV infection was 32.9 + 6.1 years, all men (Table 3). All had normal ALT values (range; 9–22 U/L). Interestingly, 5 cases were serologically positive for anti-HBs. However, only two samples had an anti-HBs level >100 IU/L (1000 IU/L and 147 IU/L in samples ID OCU 18 and OCU 43, respectively), while low levels of anti-HBs were detected in the remaining 3 samples (23.5, 12.4, 12.2 IU/L in OCU234, OCU340 and OCU243, respectively) (Table 3). T125M and Q129R substitutions were simultaneously present in only one subject positive for anti-HBs (OCU 18) (Table 3, Fig. 1b). The T125M HBsAg variant in the MHR was detected in one sample negative for anti-HBs (OCU158), while Q129R alone was detected in a further 3 samples. One of the latter was negative for anti-HBs (OCU 309) and the remaining two were positive (OCU340, 243) (Table 3, Fig. 1b). With the exception of one case all samples showed low HBV DNA level [(OCU 274), HBV DNA = 4.7 log (copy/ml)]. The viral load for the remained cases with occult HBV infection varied between 2.2-2.8 log copy/ml and 3 cases were below the detection limit of the assay. .Interestingly, the sample with the highest viral load in the occult HBV infection group exhibits no amino acid substitution in the MHR genomic region (OCU274).

**Table 3:** Descriptive analysis of cases with occult HBV infection

Sample ID	Age	ALT (IU/L)	Anti-HBs	Anti-HBs level (mIU/L)	MHR amino acid substitutions	HBV DNA (core region)	HBV DNA Log (copy/ml)
OCU234	38	22	(+)	23.5	-	(+)	2.8
OCU340	32	16	(+)	12.4	Q129R	(+)	2.7
OCU18	28	12	(+)	1000.0	T125M, Q129R	(−)	<2.0
OCU43	31	23	(+)	147.2	-	(+)	2.3
OCU243	38	9	(+)	12.2	Q129R	(+)	2.8
OCU309	45	17	(−)	0.1	Q129R	(−)	<2.0
OCU302	33	17	(−)	0.8	-	(+)	2.5
OCU274	32	12	(−)	9.3	-	(+)	4.5
OCU158	24	17	(−)	0.1	T125M	(+)	<2.0
OCU311	28	12	(−)	2.9	-	(−)	2.2

## Discussion

In this study, Occult HBV infection was detected in 22.7 % of blood donors negative for HBsAg but positive for anti-HBc, indicating that at least a quarter of cases positive for the latter had detectable HBV DNA and thus the likelihood of transmission of HBV through their blood products [14]. Said *et al.* (2013) reported an incidence of 17.2 % among a cohort similar to that of the current study [3]. Different risk factors are reported to be associated with OBI including age, male gender, anti-HBs level <100 mIU/L and positivity for anti-HBc in Egypt [3].

In Egypt, HBV screening in blood banks relies only on the detection of HBsAg. Screening for HBV by the nucleic acid amplification test (NAT) is effective in reducing the transmission of HBV via blood and blood products. In developing countries like Egypt, the high cost of the NAT may prevent its application as an essential strategy for blood-borne virus screening. Anti-HBc has been found to be an excellent indicator of the occult HBV infection and the detection of the anti-HBc [14–16] has contributed significantly in reducing the incidence of post transfusion hepatitis B [17, 18]. In this regard, measurement of anti-HBc would be more practical and may be considered as a second safeguard policy for reducing the transmission of HBV via blood products [2]. Despite the importance of anti-HBc screening for safer blood, this serological marker is not included in Egyptian blood bank screening. Then, such screening system in Egypt would miss OBI among blood donors [2–4].

The predominance of infection with HBV genotype D among cases with overt and occult HBV infection in Egypt, a previous finding supported by the present study, has allowed the analysis of S gene variants of HBV strains isolated from cases with occult and overt HBV infection who were resident in north eastern Egypt [19, 20]. The selection of samples from volunteer blood donors gives a more representative spectrum of the immune-pathological pattern of HBV infection in the general population than patients’ samples that would bias the results toward a specific immune variant of the disease [21]. Understanding the prevalence and types of HBsAg variants is of high importance, because this will affect policy decisions relating to vaccine and diagnostic reagent design.

The proportion of samples positive for anti-HBc antibody among HBsAg-negative blood donors was 12.8 %. This is higher than that previously reported anti-HBc prevalence rates among HBsAg-negative blood donors in the Mediterranean region (2.1 in Iran) [22] and the 5.6 % in Saudi Arabia [23].

Data regarding the amino acid changes of HBsAg in Egypt, particularly in the general population, are scarce. Variations in the alpha determinant region were observed in 37.8 % of strains isolated from HBsAg-positive blood donors and in 50 % of occult HBV. The incidence of HBsAg variants among random chronic carriers with HBV genotype D varied between 15 % in Morocco and 17.2 % in Iran [24]. However, Garmini *et al.* (2011) reported that the substitution rate in the MHR was 0.4 % in HBV genotype D strains isolated from HBsAg-positive blood donors in Iran [21]. Different studies in China (where genotypes C and B are prevalent) documented that mutation rates within HBsAg-positive blood donors ranged between 14.7 % in Shandong province to 50 % in Nanjing. This large difference was explained by the wider application of HBV vaccine in Nanjing [25].

Studying the underlying mechanisms of the occult HBV infection in certain poulationmay require; (1) large size studied population and (2) in vitro and in vivo experimental work to explore the virological characteristics of the detected substitutions. Despite the small number of cases with occult HBV obtained in the present study, all (with exception of one) exhibit a low viral load even in cases with MHR mutants. This finding may support the hypothesis that OBI cases are secondary to overt HBV infection and represent a residual low viremia level suppressed by strong immune response together with histological derangements occurring during acute or chronic HBV infection [22, 23].

Differences were observed between the two cohorts studied here in the type of predominant amino acid substitutions in the isolated MHR of HBsAg despite both groups originating from volunteer blood donors. Significant predominance of amino acid substitutions T125M and Q129R was observed in HBsAg isolated from occult hepatitis B cases. In the study by Candotti *et al.* (2008), T125M was detected in 11.9 % of cases with occult HBV genotype D, while none of the patients with occult HBV genotype A harbored this mutant strain [26]. The T125M substitution represents a non-conservative amino acid change which may affect the conformation of the HBsAg a determinant region and thus the binding of HBsAg-specific antibody [27]. However, one *in vitro* study indicated that this substitution failed to influence the binding of HBsAg to monoclonal or polyclonal antibodies [28].

Recent *in vitro* studies found that Q129R significantly impaired virion and/or S protein secretion in HuH7 cells and in mice is associated with lower reactivity in HBsAg assays [29]. While T125M and Q129R were the predominant HBsAg variants in these occult HBV cases, P120T and S143L were more frequent in HBsAg + blood donors. Interestingly, these substitutions were previously described in blood donors with occult HBV infection of genotype D [30].

In the present study, 29.4 % of cases positive for both anti-HBc and anti-HBs possessed detectable levels of HBV-DNA. These results are in accordance with those of Brojer *et al.* (2006) [31] and Katsoulidou *et al.* (2009) [32] who found that nearly 50 % of OBI are asymptomatic, apparently healthy blood donors carriers of anti-HBs [31, 32]. Similarly, Yotsuyanagi *et al.* (2001) found that low levels of HBV DNA was detected in the sera from 19 (38 %) of 50 HBsAg-/anti-HBc + donors. In 8 (16 %) of them, HBV did not exist as immune complexes. thus in a potentially infective form [33]. Part of this may be explained by the findings of Levicnik-Stezinar *et al.* (2008) who concluded that low levels of anti-HBs <100 mIU/L have limited neutralizing capacity [34].

## Conclusion

The profiles of the predominant amino acid substitutions different between cases with occult and overt HBV infection were detected in the alpha determinant region of the HBsAg (MHR) in blood samples from Egyptian donors. Further large population size studies are needed to explore the magnitude of occult HBV infection in Egypt and to detect HBsAg variants affecting reactivity in the HBsAg assays. The present study highlights the need for revising the strategy used for screening HBV in blood donors and blood products in Egypt. In the light of point the high cost of HBV DNA testing for all collected blood units, the present study recommends the inclusion of anti-HBc in the routine screening of blood donors in Egypt.

### Study design

#### Donors

The studied subjects in this work included two different groups of blood donors; the first group included 82 individuals who were positive for HBsAg and rejected for donation. The second group included 343 donors who were accepted for donation; being negative HBsAg, anti-HCV, anti-HIV and anti-treponema antibody. All blood donors were randomly selected from blood bank. Serum samples from the 343 HBsAg-negative donors were tested for antibodies to hepatitis B core antigen (anti-HBc). Forty four (12.8 %) of 343 HBsAg-negative blood donors were positive for anti-HBc. The latter group was further serologically tested for HBsAg antibody (anti-HBs). Finally the study included two groups of sera: one positive for anti-HBc and negative for HBsAg (n = 44), and the second group of HBsAg-positive sera (n = 82).

### Serological Markers of HBV Infection

HBsAg was determined by ELISA (Diasorin, Italy) and further confirmed by chemiluminescence enzyme immunoassay (CLEIA) (Fujirebio, Tokyo,Japan) with a detection limit of 0.05 IU/ml. Anti-HBc and anti-HBs were tested by enzyme immunoassay (EIA) (AxSYM; Abbott Japan, Tokyo, Japan). All serologic assays were carried out according to the manufacturer's instructions.

### DNA Extraction

The HBV/DNA was extracted from 200 μl of serum samples positive for HBsAg using the QIAamp DNA MiniKit (QIAGEN, Inc., Hilden, Germany), and re-suspended in 100 μl of a storage buffer provided by the kit manufacturer.

### HBV DNA Quantification

HBV-DNA was quantified by real-time detection polymerase chain reaction (RTD-PCR) primers according to the previously described protocol. The method was applied with slight modification as described previously [14, 15]. The detection limit was 100 copies/ml. The extracted samples were tested for the presence of HBV DNA by 7500 Real time PCR machine to determine the viral load by using the following primers & probe set:

- PF: 5'-CTTCATCCTGCTGCTATGCCT-3',
- PR: 5' AAAGCCCAGGATGATGGGAT-3'
- Probe: SP2: FAM-ATGTTGCCCGTTTGTCCTCTAATTCCAG-TAMRA)

The 25 μl reaction mixture volume contained 0.5 μl of each primer (10 pico mol/ul) and the probe (SB2), 6 μl water (PCR-grade), 5 μl of DNA template and 12.5 μl of the Taq polymerase. Cycling conditions were 10 min at 95 °C, 95 °C for 15 sec, and 60 °C for 1 min for 45 cycles. Fluorescence acquisition was taken once per cycle using FAM as a reporter dye and TAMARA as a quencher dye. The detection limit was 100 copies/ml.

### HBV genome amplification and sequencing

The S gene of the HBV genome (681 bp) was amplified by hemi nested PCR using specific primers. The method was applied with slight modification as described previously [14, 35]. For the first PCR (nucleotide positions 18–989):

- forward primer (IS1):5'-AAGCTCTGCTAGATCCCAGAGT-3'
- Reverse primer (HS4R): 5'- CATACTTTCCAATCAATAGG-3'

*For the second PCR (nucleotide positions 414–989):*

- Forward primer (SB1): 5'- TGCTGCTATGCCTCATCTTC-3'
- Reverse primer (HS4R): 5'- CATACTTTCCAATCAATAGG-3'

Cycling conditions were 7 min at 96 °C, 96 °C for 45 sec, and 55 °C for 45 sec and 72 °C for 45 sec for 45 cycles.

For undetectable PCR products by the previous PCR protocol, we performed hemi-nested PCR with AmpliTaq Gold® DNA Polymerase (Applied Biosystems, Waltham, MA, US). The amplicons of the genomic HBV enclosed the a determinant region of the S gene was obtained by hemi-nested PCR with forward primer HBSF2 : (5'-CTTCATCCTGCTGCTATGCCT-3' [nt 406–426]) and reverse primer HBSR2: (5'-AAAGCCCAGGATGATGGGAT-3' [nt 608–627]) for the first PCR and forward primer HBSF2 : (5'-CTTCATCCTGCTGCTATGCCT-3' [nt 406–426]) and reverse primer HBSR3. The PCR reaction was undertaken for 60 cycles (96 °C for 15 sec., 60 °C for 1 min.) followed by an extension reaction at 72 °C for 7 min.

The enhancerII/core promoter and precore regions of the HBV genome were amplified by PCR with a forward primer (IS2-2: 5′-CAT GGAGAC CAC CGT GAA CGC-3′ [nt 1607–1627]) and reverse primer (HBV1917R: 5′-CTC CAC AGA AGC TCC AAA TTCTTT A-3′ [nt 1942–1918]). PCR was initiated by the hot-star technique. The PCR reaction was undertaken for 60 cycles (96 °C for 15 sec., 60 °C for 1 min.) followed by an extension reaction at 72 °C for 7 min.

### Sequencing and Molecular Evolutionary Analysis of HBV

Amplicons were sequenced directly using the ABI Prism Big Dye ver. 3.1 kit in the ABI 3100 DNA automated sequencer (Applied Biosystems, Foster City, CA, USA). All sequences were analyzed in both forward and reverse directions with same primers used for HBV genomic amplification. HBV genotypes were determined by sequence and molecular evolutionary analysis. Reference HBV sequences were retrieved from the DDBJ/EMBL/GenBank database, aligned by CLUSTALX, and genetic distances estimated with the 6-parameter method in the Hepatitis Virus Database (http://s2as02.genes.nig.ac.jp/) [16]. Based on obtained distances, phylogenetic trees were constructed by the neighbor-joining (NJ) method with the mid-point rooting option. To confirm the reliability of the phylogenetic trees, bootstrap resampling tests were performed 1000 times for analysis by the ODEN program of the National Institute of Genetics. The deduced amino acid sequences of the HBsAg a-determinant regions in the donor HBV strains were compared to the HBV genotype D reference strains as proposed previously [17]; and to the amino acid sequences as present in the public domain databases, using the protein–protein Blast utility [18]. The nucleotide sequences data described in this paper will appear in the DDBJ/EMBL/GenBank sequences database with accession numbers; AB981241-AB981313 and AB981171-AB981180.

### Statistical analysis

Statistical analysis was performed with the Fisher’s exact probability test and the independent t-test for the continuous variables using the SPSS software package (SPSS, Chicago, IL, USA). *P*-values (two-tailed) less than 0.05 were considered to be statistically significant.

### Ethical consideration

This study was approved by research ethics committee, Faculty of Medicine, Suez Canal University (No. 973)

### Ethics, consent and permissions

Informed written consent was taken from the patients under study. The confidentiality of any information pertaining to the patients was assured. No obligation on the patients to participate in the study.

### Consent to publish

We have obtained written consent from all participants to publish their data.
